# Brain Activation in Contrasts of Microexpression Following Emotional Contexts

**DOI:** 10.3389/fnins.2020.00329

**Published:** 2020-04-29

**Authors:** Ming Zhang, Ke Zhao, Fangbing Qu, Kaiyun Li, Xiaolan Fu

**Affiliations:** ^1^Institute of Psychology, Chinese Academy of Sciences, Beijing, China; ^2^College of Preschool Education, Capital Normal University, Beijing, China; ^3^School of Education and Psychology, University of Jinan, Jinan, China; ^4^Department of Psychology, University of Chinese Academy of Sciences, Beijing, China

**Keywords:** emotion context, microexpression, recognition, activation, fMRI

## Abstract

The recognition of microexpressions may be influenced by emotional contexts. The microexpression is recognized poorly when it follows a negative context in contrast to a neutral context. Based on the behavioral evidence, we predicted that the effect of emotional contexts might be dependent on neural activities. Using the synthesized microexpressions task modified from the Micro-Expression Training Tool (METT), we performed an functional MRI (fMRI) study to compare brain response in contrasts of the same targets following different contexts. Behaviorally, we observed that the accuracies of target microexpressions following neutral contexts were significantly higher than those following negative or positive contexts. At the neural level, we found increased brain activations in contrasts of the same targets following different contexts, which reflected the discrepancy in the processing of emotional contexts. The increased activations implied that different emotional contexts might differently influence the processing of subsequent target microexpressions and further suggested interactions between the processing of emotional contexts and of microexpressions.

## Introduction

As we know, emotional information always affects the recognition of subsequent facial expression and then exerts an important context effect (Wieser and Brosch, 2012). It will facilitate the recognition of subsequent facial expressions if they convey the same emotional components (Werheid et al., 2005). For example, anger is recognized more accurately following a negative context, whereas happiness is recognized better following a positive context (Hietanen and Astikainen, 2013). The microexpression, as a quick facial expression, generally lasts for 1/25 to 1/5 s and occurs in the flow of facial expressions, especially when individuals try to repress or conceal their true emotions (Ekman, 2009). Its recognition is influenced by emotional stimuli (e.g., facial expression) appearing before and after the microexpressions (i.e., the emotional contexts). Microexpressions are recognized poorly when they followed a negative context, regardless of the duration of the microexpressions (Zhang et al., 2014). However, existing studies provide limited behavioral evidence for the presence of an effect of emotional context in microexpression recognition (Zhang et al., 2014, 2018). In order to recognize microexpressions more accurately in realistic emotional contexts, further evidence for the neural basis of the effect deriving from the emotional context is necessary.

The effect of emotional contexts on the perception of facial expressions could be reflected in neural activations (Schwarz et al., 2013). The facial expression alone generally activated visual processing regions, yet the facial expression with a context was more associated with social and emotional processing regions (Lee and Siegle, 2014). Emotional contexts including some affective stimuli could influence cerebral cortex reactions, altering activation regions or activation levels. Facial expressions conveying specific emotions engage specific brain areas, such as the medial prefrontal cortex, the fusiform gyrus, the superior temporal gyrus, the parahippocampal gyrus, the insula, the precuneus, the inferior parietal, and the amygdala (Haxby et al., 2000; Heberlein et al., 2008). Moreover, brain activities related to facial expressions are not always clear cut and are strongly influenced by the emotional context. Facial expressions will be interpreted differently in various emotional contexts (Schwarz et al., 2013). By presenting target (fearful/neutral) faces against the background of threatening or neutral scenes, Van den Stock et al. (2014) found that the emotional valence of contexts modulates the processing of faces in the right anterior parahippocampal area and subgenual anterior cingulate cortex, which showed higher activations for targets in neutral contexts compared to those in threatening contexts. In addition, response inhibitions coming from the interaction of facial expressions and preceding contexts were observed in the left insula cortex and right inferior frontal gyrus (Schulz et al., 2009). Consistent with these accounts, brain responses to ambiguous facial expressions (surprise) were found to be modified by contextual conditions—that is, activations (especially in the amygdala) were stronger for surprised faces embedded in negative contexts compared to those in positive contexts (Kim et al., 2004). These findings showed that the perception of facial expression is modulated by contextual information, reflecting context-dependent neural processing of facial expressions.

In view of the effect of emotional context on the brain’s responses to facial expressions, microexpressions should be influenced by emotional contexts. Behavioral evidence for the effect of emotional contexts on microexpression recognition leads us to believe that the effect of emotional contexts should depend on neural activities. The present fMRI study focused on brain activation in contrasts of the microexpression following different emotional contexts and aimed to provide neural evidence for the potential effect of emotional context on microexpressions. The previous study showed that emotion recognition is modulated by a distributed neural system (Zhao et al., 2017). The process of emotion recognition involves increased activity in visual areas (e.g., fusiform gyrus), limbic areas (e.g., parahippocampal gyrus and amygdala), temporal areas (e.g., superior temporal gyrus and middle temporal gyrus), and prefrontal areas (e.g., medial frontal gyrus and middle frontal gyrus) (Haxby et al., 2000; Heberlein et al., 2008). Based on these findings, it is reasonable to predict that contrasts of the same targets following different contexts will elicit different patterns of increased brain activity. This study adopted a synthesized task modified from the Micro-Expression Training Tool (METT) to simulate a microexpression (Ekman, 2002; Shen et al., 2012) and compared the brain activations of contrasts of the same targets following different contexts.

## Materials and Methods

### Participants

Twenty-one healthy, right-handed undergraduates (age ranged from 18 to 23, *M* = 20.90, *SD* = 1.37; 11 females) with normal or corrected-to-normal vision participated in our fMRI study and were compensated for their participation. Before entering the MRI scanner, they completed a questionnaire provided by the Southwest University MRI Centre that required all individuals to report honestly their current health status and medical records, including physical injuries and mental disorders. No participant reported a neurological or psychiatric history. Written informed consent to participate was obtained, and participants were informed of their right to discontinue participation at any time. The experimental protocol was approved by the Institutional Review Board of the Institute of Psychology at the Chinese Academy of Sciences. All procedures were conducted according to the Declaration of Helsinki.

### Stimuli

**FIGURE 1:**
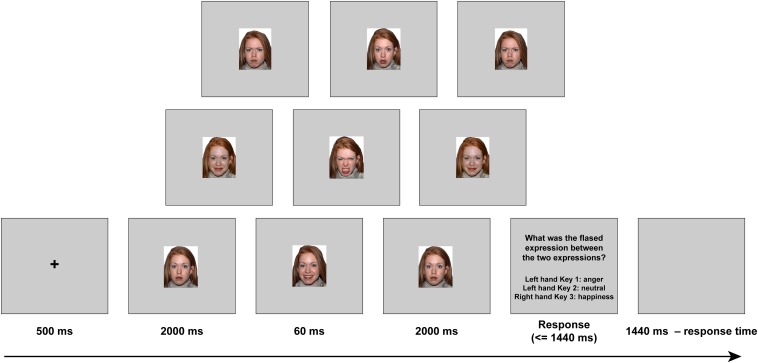
The experimental setup of each trial.

The materials, including 120 images (20 models, 10 females), were adapted from a previous study (Zhang et al., 2018). The visual stimuli were presented via a video projector (frequency, 60 Hz; resolution, 1,024 × 768; frame-rate, ∼16.7 ms) onto a rear-projection screen mounted at the head of the scanner bore (see stimuli samples in Figure 1). Participants viewed the stimuli through a mirror on the head coil positioned over their eyes. All the stimuli (visual angle, 11.8° × 15.1°) were displayed on a uniform silver background.

### Procedure

The task was adopted from the previous study (Zhang et al., 2014) and was modified for the present fMRI experiment. Both stimulus presentation and behavioral response collection were controlled by E-Prime 2.0 (Psychological Software Tools, Inc., Pittsburgh, PA, United States). Participants performed a practice experiment outside the MRI, using the same procedure as the real experiment. There were four sessions in total, each lasting 8 min. Each session included nine experimental conditions, in which three emotional contexts (negative, neutral, and positive) and three target microexpressions (anger, neutral, and happiness) were randomly combined, as well as a blank condition. All of these conditions were repeated eight times—that is, each of these nine conditions was repeated 32 times in total in four sessions. The trial sequence in each session was randomized with a trial time of 6 s.

Each trial proceeded as follows (see Figure 1). First, a black fixation cross was presented for 500 ms, followed by either an angry, neutral, or happy expression context (all with closed mouth) for 2,000 ms (119 frames). Subsequently, one of the three target microexpressions (anger, happiness, or neutral, all with open mouth, the same model as in the forward context) was presented for 60 ms (four frames). Then, the same expression context was presented for 2,000 ms (119 frames) again. After that, the task instructions were presented, and participants were asked to recognize the fleeting expression by pressing one of the three buttons (half of the participants were told to press 1 or 2 with the right hand, and 3 with the left hand, while the other half of the participants were told to press 1 or 2 with the left hand, and 3 with the right hand). If there was no reaction, the task instructions would disappear after 1,440 ms. Finally, a blank screen was presented for 1,440 ms minus reaction time to ensure that the total duration of each trial was the same.

### Data Acquisition

A Siemens 3.0-T scanner (Siemens Magnetom Trio Tim, Erlangen, Germany), equipped with a 12-channel head matrix coil, was used for functional brain imaging in the present study. The participant’s head was securely but comfortably stabilized with firm foam padding. Scan sessions began with shimming and transverse localization. Functional data were acquired using an echo-planar imaging (EPI) sequence using an axial slice orientation [33 slices, repetition time (TR)/echo time (TE) = 2,000/30 ms, slice thickness = 3 mm, field of view (FOV) = 200 mm, flip angle = 90°; matrix size, 64 × 64] covering the whole brain. A high-resolution T1-weighted 3D MRI sequence was acquired between the second and third sessions of fMRI (ascending slices, 128 slices, TR/TE = 2,530/2.5 ms, FOV = 256 mm × 256 mm, flip angle = 7°, voxel size = 1.0 mm × 1.0 mm × 1.3 mm).

### Data Analysis

The data were preprocessed and analyzed using Statistical Parametric Mapping software SPM8 (Wellcome Department of Cognitive Neurology, London, United Kingdom). Standard fMRI preprocessing was performed including slice timing, realignment (data with translation of more than 3 mm or rotation angle greater than 2.5° were removed), spatial normalization [EPI template; Montreal Neurologic Institute (MNI)], reslicing (3 mm × 3 mm × 3 mm voxels), and smoothing with a 6-mm full-width at half-maximum (FWHM) Gaussian kernel. The conventional two-level approach using SPM8 was adopted for event-related fMRI data. The variance in blood-oxygen-level-dependent (BOLD) signal was decomposed in a general linear model separately for each run (Friston et al., 1994). The time course of activity of each voxel was modeled as a sustained response during each trial, convolved with a standard estimate of the hemodynamic impulse response function (Boynton et al., 1996). Low-frequency BOLD signal noise was removed by high-pass filtering of 128 s. For the whole-brain analysis, cluster-level familywise error (FWE) corrected at *p* < 0.05 and cluster size ≥ 13 voxels were applied. Considering the number of missed trials without a response was minor (56 trails out of the total of 6,048), we kept all the trials for the next data processing.

The whole-brain analysis was conducted to reveal the brain activation using context and target as explanatory variables. The initial comparisons of task-related events^1^ time-locked to the front context onset (duration = 2.06 s) and baseline were performed by a single-sample *t*-test in the first-level analysis (Fusar-Poli et al., 2009; Sabatinelli et al., 2011). In the second-level analysis, using the context-by-target interaction term (e.g., negative context–anger target), we analyzed the brain activation related to task-related conditions. The emotional reactivity contrasts^2^ were obtained by group analysis in second-level analysis using paired *t-*test (*p* < 0.001).

## Results

### Behavioral Performance

**FIGURE 2:**
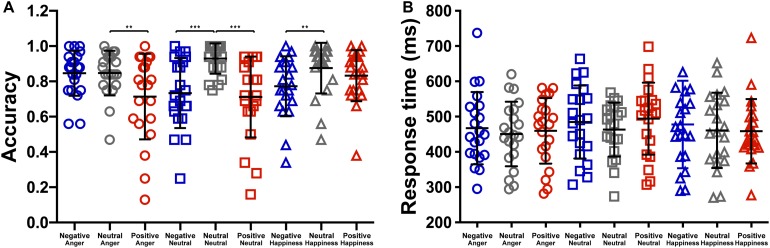
The effect of context on the **(A)** accuracy and the **(B)** response time (***p < 0.001, **p < 0.01).

The effect of emotional context on behavioral measures was assessed by applying a two-way repeated ANOVA to the recognition accuracies, with the context and the target as within-participant variables. It revealed a significant main effect of context, *F*(2,40) = 33.76, *p* < 0.001, $\eta_{\text{p}}^{2}$ = 0.628. The accuracies of targets following negative and positive conditions were significantly lower than that following neutral condition (see Table 1), *t*(19) = −5.88, *p* < 0.001, *d* = 0.27; *t*(19) = −7.71, *p* < 0.001, *d* = 0.35. The main effect of target microexpression was not significant, *F*(2,40) = 0.54, *p* = 0.587. The interaction of context and target reached significance, *F*(4,80) = 4.58, *p* = 0.002, $\eta_{\text{p}}^{2}$ = 0.186. Further analysis revealed that the accuracy rate for anger was significantly higher following neutral context than that following positive context, *t*(19) = 3.35, *p* = 0.009, *d* = 0.69; the accuracy rate for neutral was significantly higher following neutral context than that following negative or positive context, *t*(19) = 4.90, *p* < 0.001, *d* = 1.29; *t*(19) = 4.87, *p* < 0.001, *d* = 1.27; the accuracy rate for happiness was significantly higher following neutral context than that following negative context, *t*(19) = 3.43, *p* = 0.008, *d* = 0.66 (see Figure 2A).

**TABLE 1:** Means and standard deviations of accuracies in all conditions.

	Context			Mean accuracies
	Negative	Neutral	Positive	
	*M* ± SD	*M* ± SD	*M* ± SD	
**Target**				
Anger	0.84 ± 0.13	0.85 ± 0.13	0.71 ± 0.24	0.80 ± 0.40
Neutral	0.73 ± 0.20	0.93 ± 0.09	0.71 ± 0.23	0.79 ± 0.41
Happiness	0.77 ± 0.17	0.87 ± 0.14	0.83 ± 0.15	0.83 ± 0.38
Mean accuracies	0.78 ± 0. 41	0.88 ± 0.32	0.75 ± 0.43	

We also analyzed the response time to examine the effect of emotional context on target. The two-way repeated ANOVA showed a significant main effect of context, *F*(2,40) = 3.988, *p* = 0.026, $\eta_{\text{p}}^{2}$ = 0.166. The response time of targets following negative condition (476.40 ± 213.35) were marginally significantly longer than that following neutral condition (458.11 ± 195.37), *t*(19) = 2.45, *p* = 0.07 (see Figure 2B). The main effects of target microexpression and the interaction were not significant, *F*(2,40) = 2.36, *p* = 0.108; *F*(4,80) = 0.94, *p* = 0.446.

### fMRI Results

**FIGURE 3:**
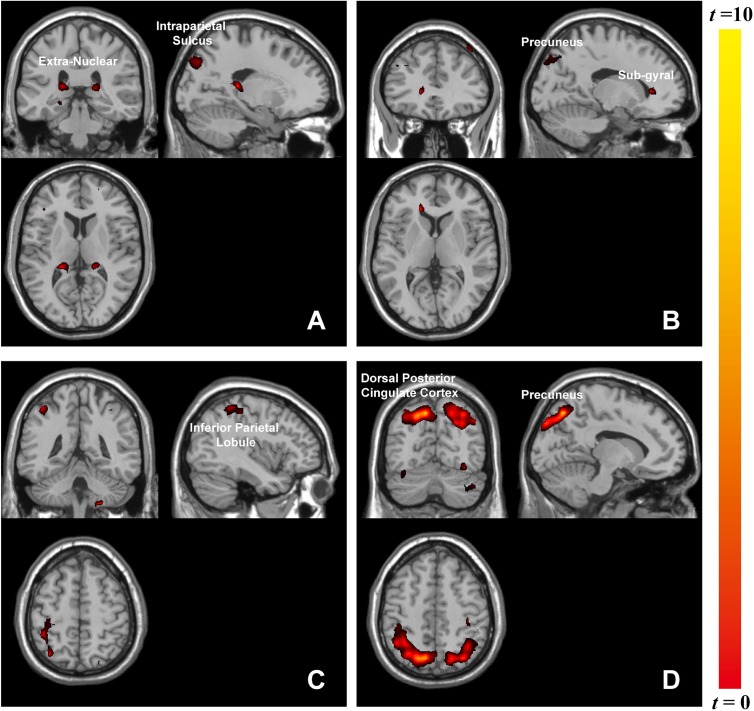
Brain activation in contrasts of microexpressions following emotional contexts. When target was anger: **(A)** negative context > neutral context, extranuclear (*x* = 27, *y* = –39, *z* = 15), intraparietal sulcus (*x* = 18, *y* = –84, *z* = 36), **(B)** positive context > neutral context, precuneus (*x* = 24, *y* = –75, *z* = 39), subgyral (*x* = 33, *y* = –78, *z* = –6); when target was neutral: **(C)** negative context > neutral context, inferior parietal lobule (*x* = 42, *y* = –42, *z* = 54), **(D)** positive context > neutral context, precuneus (*x* = 12, *y* = –72, *z* = 48), dorsal posterior cingulate cortex (*x* = –18, *y* = –84, *z* = 36).

The whole-brain analysis based on paired *t*-test model for contrast conditions revealed that brain activations to target microexpressions varied across emotional contexts. Several areas exhibited significant increases in BOLD signals for contrasts of the same targets following different contexts (see Table 2). When the target was anger, there were increased BOLD signals mainly in the right intraparietal sulcus and extranuclear for the contrast of negative context against neutral context (negative context–anger target > neutral context–anger target, Figure 3A) whereas in the right precuneus and subgyral for the contrast of positive context against neutral context (positive context–anger target > neutral context–anger target, Figure 3B). When the target was neutral, there were increased BOLD signals mainly in the right inferior parietal lobule for the contrast of negative context against neutral context (negative context–neutral target > neutral context–neutral target, Figure 3C) whereas in the right precuneus and left dorsal posterior cingulate cortex for the contrast of positive context against neutral context (positive context–neutral target > neutral context–neutral target, Figure 3D; see Supplementary Table S1 for more results).

**TABLE 2:** Coordinates in Montreal Neurologic Institute (MNI) space and associated *t* scores showing the BOLD differences for the contrast of emotional contexts followed by the same microexpressions.

Brain regions	BA	Cluster size	*t*	*Z*	MNI		
					*x*	*y*	*z*
**Target anger: negative > neutral**							
Intraparietal sulcus (R)	7	100	5.26	4.00	18	−84	36
Extranuclear (R)		55	5.79	4.25	27	−39	15
**Target anger: positive > neutral**							
Precuneus (R)		144	4.78	3.76	24	−75	39
Subgyral (R)		49	5.95	4.31	33	−78	−6
**Target neutral: negative > neutral**							
Inferior parietal lobule (R)		111	6.00	4.34	42	−42	54
**Target neutral: positive > neutral**							
Precuneus (R)		1,108	8.31	5.19	12	−72	48
Dorsal posterior cingulate cortex (L)	31	772	8.47	5.23	−18	−84	36
Cerebellum_10 (L)		281	5.57	4.15	−24	−30	−42
Inferior semilunar lobule (L)		151	5.45	4.09	−30	−78	−45
Declive (R)		105	5.17	3.95	39	−60	−21

**x*, *y*, and *z* are coordinates in the MNI space. All *p*-values (*p* < 0.05) passed familywise error (FWE) correction at cluster level.*

## Discussion

We verified that emotional contexts influence microexpression recognition, which is consistent with previous findings (Zhang et al., 2014). Target microexpressions were recognized better following neutral contexts than those following positive or negative contexts. Emotional stimuli affect how we process and respond to targets (Siciliano et al., 2017). Compared to the neutral stimuli, the emotional ones can be highly salient, and these emotionally salient events can disrupt the recognition of targets (Siciliano et al., 2017). Attention allocation was reported to be related to and modulated by the emotional valences of stimuli—that is, emotional stimuli could capture more attention (Wilson and Hugenberg, 2013). Increasing attentional load decreases the processing resources available for the subsequent task (Kurth et al., 2016). In our study, it seemed that there was not enough attention directed to the subsequent target microexpressions because of emotional contexts, and poor performance for recognition was therefore observed. Our fMRI results also supported this: there were increased activities in some attention-related functional regions when microexpressions followed negative or positive contexts.

Emotional stimuli, either pleasant or unpleasant, prompted significantly more activity than did neutral pictures (Lang et al., 1998). Accordingly, we found that brain activities associated with the same target microexpression following various emotional contexts differed in functional regions. Anger microexpressions followed negative context (negative context–anger target) compared to neutral context (neutral context–anger target) in that they activated the right intraparietal sulcus and extranuclear, whereas they followed positive context (positive context–anger target) compared to neutral context (neutral context–anger target), activating the right precuneus. Furthermore, it was observed that different regions responded to the neutral-target-related emotional contrast, for instance, the right precuneus. As in previous studies, these regions played a role in facial expression recognition (Mourao-Miranda et al., 2003). In our study, positive context compared to neutral context with the same target activated more brain regions, including the right precuneus. The precuneus participated in positive stimuli assessment (Paradiso et al., 1999), memory (Berthoz, 1997), and attention (Goldin and Gross, 2010). Unlike previous findings that negative stimuli (fear expressions) could also significantly activate the emotion-related areas (Sprengelmeyer et al., 1998), here, we only found that positive context activated the right precuneus, meaning that positive emotions could cause strong emotional arousal during this microexpression task. The left extranuclear was also reported to be significantly activated by happy faces compared with neutral faces (Trautmann et al., 2009) and was related to emotional regulation (McRae et al., 2008). Here, we found that the right extranuclear was activated in the negative context compared to the neutral context when they were followed by anger microexpressions, implying that the activities of facial expressions including context and target could be complicated. The brain responses in these contrasts reflected the discrepancy in the processing of emotional contexts, which could suggest interactions between the processing of emotional contexts and of microexpressions. These discrepancies in emotional contexts implied that different emotional contexts might differently influence the processing of subsequent target microexpressions.

Based on the findings on behavioral performance and brain activities, emotional contexts lead to a decrease in recognition accuracies and an increase in context-related activations in some emotional and attentional regions. The increased perceptual load of negative and positive contexts yields increased brain activations along with decreased behavioral performance, due to the additional monitoring and attention necessary for inhibition of emotional contexts (Siciliano et al., 2017). Thus, the recognition of microexpression would be affected by the emotional contexts, which has been proven on behavioral performance. These activities in attention-related regions indicated that attention being occupied by negative and positive contexts might be a source of the effect of emotional contexts on the processing of microexpressions.

### Limitations

Considering that a microexpression is very fast and is always submerged in other microexpressions, we did not leave a long break between context and target in order to simulate the real situation in which the microexpression happened. This led to our being unable to extract the exact BOLD response to the target and instead having combined the forward context and target and examined the whole duration. Here, our findings only showed that there were discrepancies in brain response between contrasts of the same targets following different contexts and suggested a limited potential effect of emotional context on subsequent target microexpressions, but not a very exact effect on microexpressions. Taking these issues into account, future work could focus on exploring the processing of different functional areas’ responses to microexpression with more ecological validity and suitable experimental design in order to explore the neural mechanism for the effect of emotional context on microexpression.

## Conclusion

Compared with previous studies on emotional processing, our study made a bold attempt to explore the context effect on microexpression using the unconventional fMRI paradigm. The study showed that there were discrepancies between contrasts of the same targets following different contexts and suggested interactions between the processing of emotional contexts and of microexpressions. That is, brain responses in these contrasts reflected discrepancy in the processing of emotional contexts, meaning that different emotional contexts might differently interfere with the processing of subsequent target microexpressions.

## Data Availability Statement

All datasets generated for this study are included in the article/Supplementary Material.

## Ethics Statement

The studies involving human participants were reviewed and approved by the Institutional Review Board of the Institute of Psychology at the Chinese Academy of Sciences. The patients/participants provided their written informed consent to participate in this study.

## Author Contributions

MZ, KZ, and XF contributed to designing the experiment, analyzing the data, and writing the manuscript. FQ contributed to analyzing the data and writing the manuscript. MZ and KL contributed in collecting the data.

## Conflict of Interest

The authors declare that the research was conducted in the absence of any commercial or financial relationships that could be construed as a potential conflict of interest.

## Footnotes

1 The nine task-related events (negative context–anger target, neutral context–anger target, positive context–anger target, negative context–neutral target, neutral context–neutral target, positive context–neutral target, negative context–happiness target, neutral context–happiness target, and positive context–happiness target).

2 Negative context–anger target > neutral context–anger target, positive context–anger target > neutral context–anger target, negative context–neutral target > neutral context–neutral target, positive context–neutral target > neutral context–neutral target, negative context–happiness target > neutral context–happiness target, and positive context–happiness target > neutral context–happiness target.
