# Supplementary material for: Brain Activation in Contrasts of Microexpression Following Emotional Contexts
Source: Front Neurosci. 2020 Apr 29;14:329. doi: 10.3389/fnins.2020.00329 (PMC7202324; doi:10.3389/fnins.2020.00329)
Supplement: Supplementary file 1 [file Table_1.DOCX]

**Table** Coordinates in MNI space and associated *t*-scores showing the increased BOLD for the contrast of neutral contexts and emotional context followed by the same micro-expressions

| **Brain regions** | **BA** | **Cluster**  **size** | ***t*** | ***Z*** |  | **MNI** |  |
| --- | --- | --- | --- | --- | --- | --- | --- |
|  |  |  |  |  | **x** | **y** | **z** |
| **Target neutral: positive < neutral** |  |  |  |  |  |  |  |
| Brodmann Area 19 (L) | 19 | 94 | 10.49 | 5.78 | –42 | –90 | 3 |
| Brodmann Area 19 (R) | 19 | 86 | 7.58 | 4.95 | 42 | –90 | 3 |
| Brodmann Area 23 (R) | 23 | 52 | 5.93 | 4.31 | 3 | –60 | 18 |
| Cuneus (L) |  | 87 | 5.31 | 4.02 | –9 | –90 | 18 |
| **Target positive: negative < neutral** |  |  |  |  |  |  |  |
| Superior occipital gyrus (L) |  | 226 | 8.85 | 5.35 | –15 | –90 | 39 |
| Brodmann Area 19 (R) | 19 | 218 | 6.64 | 4.60 | 18 | –87 | 39 |
| Brodmann Area 24 (L) | 24 | 80 | 5.95 | 4.32 | –3 | –6 | –27 |
| Insula (L) |  | 61 | 5.40 | 4.07 | –42 | 3 | 3 |
